# Enhancing Class Culture: Assessing and Improving the Impact of the "Thriving Together" Workshop for Dalhousie Medical Students

**DOI:** 10.7759/cureus.72478

**Published:** 2024-10-27

**Authors:** Brett Henderson, Tyler Herod, Emma McDermott

**Affiliations:** 1 Faculty of Medicine, Dalhousie University, Halifax, CAN; 2 Department of Health Research Methods, Evidence, and Impact, McMaster University, Hamilton, CAN

**Keywords:** class dynamics, collaborative learning, dalhousie university, medical education, medical school culture, quality improvement, student-led initiatives, thriving together workshop

## Abstract

Introduction

Collaboration and collegiality in medical school benefit students’ experiences and contribute to improved patient care. Learning environments have the potential to foster competition and discourage collaboration. Thriving Together was created to address class dynamics and culture early in medical training.

Objective

The objective of the study is to thoroughly evaluate the Thriving Together Workshop.

Methods

The Thriving Together workshop, led by upper-year students, comprises a presentation on class culture, anonymous polling, and small-group case-based exercises. It concludes with a large-group discussion. Pre- and post-workshop survey results were collected via Opinio software. A basic statistical and thematic analysis was conducted to identify response themes.

Results

The post-workshop survey response rate was 29 out of 41 attendees (70.7%) in 2022 and 20 out of 55 attendees (36.4%) in 2023. Forty-eight (96.6%) respondents would recommend the workshop to next year’s medical cohort, and 44 (89.8%) were interested in a follow-up workshop. Qualitative comments were positive, with feedback focused on attendance, group randomization, and the need for formal resources and post-workshop follow-up.

Conclusion

The Thriving Together workshop has a positive impact on class culture as evidenced by voluntary attendance and positive survey responses. Strategies to improve attendance will be implemented for upcoming sessions and will focus on refining the workshop to encourage inter-group interactions. In addition, formal resources will be provided to those interested. These adjustments aim to sustain the positive impact of the Thriving Together initiative on medical school culture.

## Introduction

It is well known that medical schools select students for qualities like discipline, ambition, and organization. Admission requirements for Canadian medical schools are at an all-time high, forcing applicants to strive for near-perfect marks while balancing a variety of extracurriculars with their remaining free time. It is perhaps not surprising, therefore, that the medical school learning environment fosters competitiveness; students continue to strive for new ways to stand out amongst their peers. In a recent study, students cited an absence of social support networks as a primary contributor to challenges they experienced in their transition to medical school [1].

From a general health perspective, higher levels of social cohesion within a community are correlated with lower mortality rates and result in reported improved well-being [2,3]. This is no different for medical students, who report collegiality between peers and a sense of belonging as protective factors [4-6]. Furthermore, collaboration and collegiality in medical school were found to have positive benefits on students’ confidence, clinical skills, and evaluations [5,7,8]. These benefits also extend into the professional development of medical students and in turn, the impact they will have on their own patients in their future careers. A higher level of group cohesion was found to have a positive relationship with self-directed learning within the same group, overall fostering the development of lifelong learners [7]. Additionally, there is consensus that interprofessional collaborative care in healthcare settings improves patient outcomes, satisfaction and access to care, reduces admissions and staff turnover, and is overall key to improving our health system [9,10].

While there has been longstanding debate on how best to select students for medical school [11], schools often aim to find candidates that show traits such as resilience, discipline, and ambition, among other AAMC core competencies [12]. Nonetheless, the admission process itself, and the years of academic and extracurricular preparation beforehand to build a strong application, often creates an inherent competitiveness amongst medical school classmates. Studies have also shown that student demographics often underrepresented in medical school classes (e.g. those from lower socioeconomic backgrounds, minorities, the LGBTQIA+ community, newcomers, and first-generation medical students) struggled to fit into medical school more than their peers [13-16]. In one study of 5000 Florida medical students, second-year students ranked “competition with peers” as the largest factor contributing to their stress in medical school [17]. While medical schools have begun to focus on training related to interprofessional communication [18], medical students are often given little to no formal training on interpersonal conflict resolution [19] or leadership [20] to help them combat these challenges.

In the summer of 2021, under the Dalhousie University Faculty of Medicine’s “DalMedWell” programming, a working group of medical students with representation from the Classes of 2021-2024 was formed to discuss class culture. Discussions revolved around group norms, class identity, and shared values within each medical school class, what barriers exist for class cohesion and support, and how to better equip future classes to build a strong community within cohorts. This working group identified gaps in the formal curriculum and decided to develop the Thriving Together Workshop.

Thriving Together is a two-hour student-led workshop to support the incoming Dalhousie University medical school class by openly addressing class dynamics, culture, and expectations in medical school and equip the incoming cohort with the building blocks to create their class community. The workshop has been offered in late October for the past three years, allowing students to develop relationships after their initial orientation week and avoid major exam periods. The workshop is offered in person at both the Halifax, Nova Scotia campus and the Saint John, New Brunswick satellite campus. Attendance is optional, and dinner is offered as an incentive to attend. Communications are delivered via email, social media (Facebook, Instagram), and short presentations before lectures to encourage attendance. The initial reception of the workshop was positive [21] and, because of this, Thriving Together has become an annual workshop for first-year Dalhousie Medical Students. This workshop remains a student-led initiative.

This paper aims to identify areas of improvement and provide recommendations for the Thriving Together Workshop based on participant feedback to ensure future workshops can continue to support medical students.

## Materials and methods

Ethics approval

As this was a quality improvement study and did not utilize identifiable personal data, Dalhousie Research Ethics Board approval was not required [22].

Workshop development and facilitation

**Figure 1:**
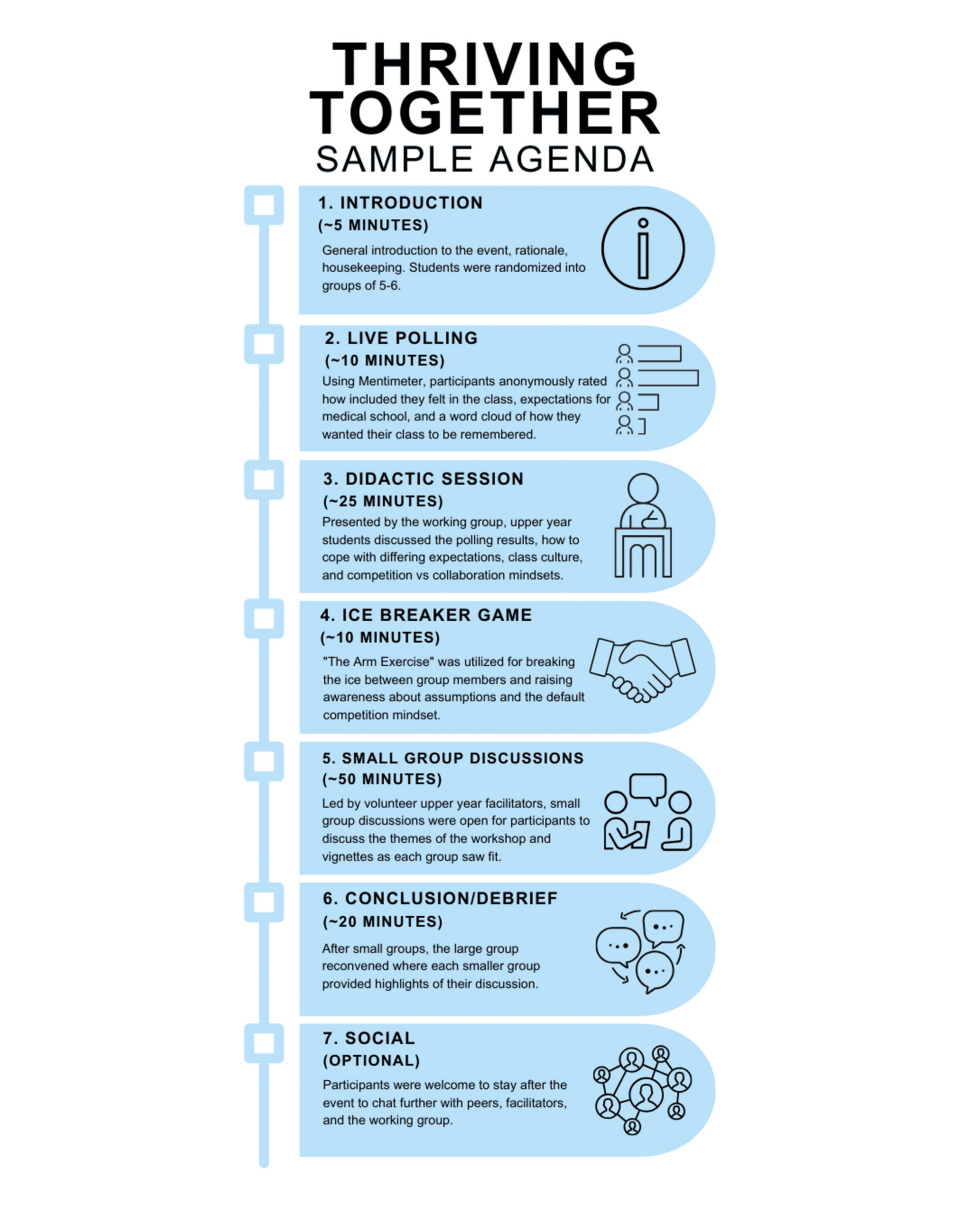
Sample agenda of the two-hour Thriving Together workshop

The structure of the Thriving Together workshop was developed by the working group. A sample agenda is shown in Figure 1.

Prior to the workshop, students completed an anonymous pre-survey exploring their expectations for medical school, their perceived sense of belonging within the class, and their perceived importance of class culture within their overall medical school experience. Google Forms was utilized to collect attendance information and anonymous pre-survey responses. These anonymized results were presented to students during the opening didactic session of the workshop.

Upon arrival at the workshop, first-year medical students were randomized into groups of five to six students and two upper-year facilitators. Randomization was used to ensure that social circles and friends did not migrate to the same tables. An anonymous polling session allowed students to respond honestly to questions about their experiences and perspectives while allowing attendees to see the live responses on the screen. Participants were aware that all discussions that took place in the room were to be treated as confidential and any notes were shredded at the end of the workshop.

**Figure 2:**
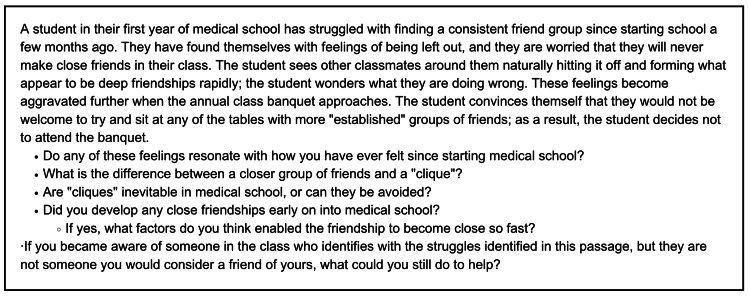
A sample vignette developed by the Thriving Together Working Group used during workshops in 2022 and 2023.

**Figure 3:**
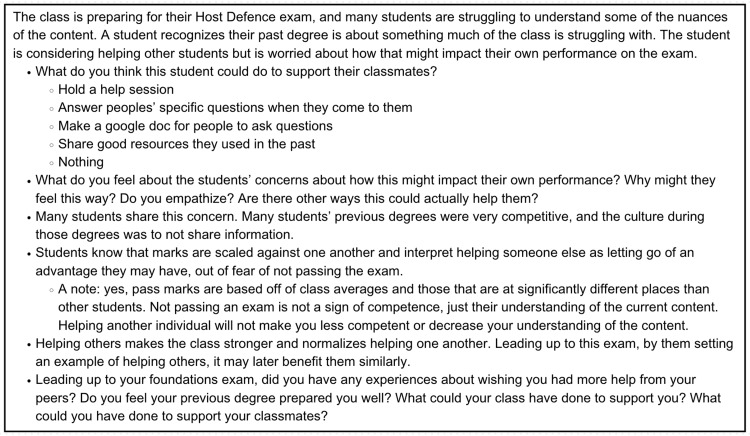
A sample vignette developed by the Thriving Together Working Group used during workshops in 2022 and 2023.

The Thriving Together working group (composed of upper-year medical students) drew on personal experiences to create 20 vignettes highlighting scenarios that may be encountered during medical school. Vignettes were printed and distributed to each table to be used to help stimulate initial discussion. Topics discussed in vignettes included student belonging, academic performance, satellite campus dynamics, collaboration and teamwork within the class, and dealing with uncertainty. Sample vignettes are displayed in Figures 2, 3.

Data collection and analysis

Following the 2022 and 2023 workshops, students were sent a post-workshop survey (see appendix) to gauge their perception of class culture and identify any areas of improvement for future workshops. The survey was developed and reviewed by the Thriving Together Working Group and delivered to students via Opinio Survey software (ObjectPlanet, Oslo, Norway). Using established interpretive description methodology [23], narrative responses were first grouped by one investigator (B.H), then reviewed by other investigators, and revised until all investigators were satisfied. Descriptive statistics were used for the analysis of quantitative results. Qualitative responses were coded into categories and themes.

## Results

Although each year’s workshop was held at both Dalhousie’s primary campus (Halifax, Nova Scotia) and its satellite campus (Saint Johns, New Brunswick), all presented data pertains only to the Halifax campus due to insufficient response rates from the satellite campus.

**Figure 4:**
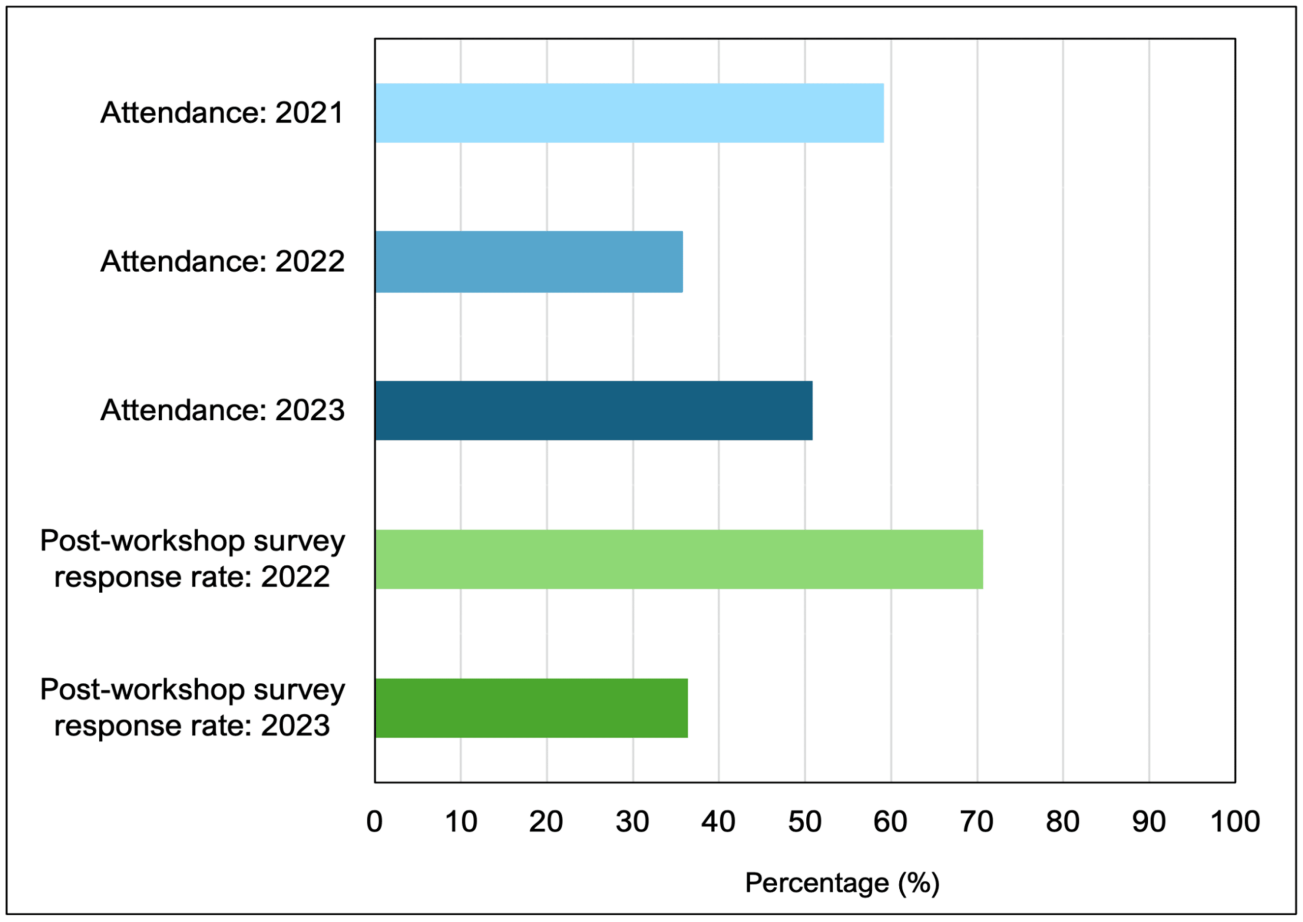
The attendance for each workshop and the post-workshop survey response rates (all expressed as percentages)

The attendance for each workshop is shown in Figure 4. Calculated as a percentage of class size, the attendance was 59.2% (50/98) in 2021, 35.7% (41/115) in 2022, and 50.9% (55/108) in 2023, averaging 48.6% overall. Post-workshop survey response rates are also shown in Figure 4. The workshop in 2021 did not collect formal responses following the workshop, so qualitative results are only included from 2022 and 2023. Response rates were 70.7% (29/41) in 2022 and 36.4% (20/55) in 2023, averaging 53.5%.

For the 2022 survey, of the 29 respondents, 23 (79.3%) felt that the workshop met their expectations, 28 (96.6%) felt that there were no negative effects of attending Thriving Together, 27 (93.1%) were interested in a follow-up session in the Spring, and 28 (96.6%) would recommend the workshop to next year’s medical cohort. Meeting peers and having upper-year facilitators contributing to small-group discussions were highlighted as the main benefits of the workshop. When asked what was missing from the workshop, patterns in responses included reference to more participation from the class to lend to a fuller discussion (Table 1), distributing post-workshop resources regarding how to effectively make friends and improve class culture, and having an expert in workplace culture speak to the class. When asked who should coordinate further support regarding class cohesion and effectiveness, 22 (75.9%) chose informal class leaders, 20 (69.0%) chose class council, and 14 (48.3%) chose the Dalhousie Medical Student Society. In terms of how future support should be provided, the most favorable option was facilitated small group sessions, with 20 (69.0%) selecting this option.

**Table 1:** Quotes obtained from both the 2022 and 2023 post-workshop surveys illustrating key areas of improvement

Themes for Improvement	Illustrative Survey Quotes
Attendance	“I wish there were more people in my class here that way we might’ve had a more full discussion with people I don’t normally have deeper discussions with.” “I would have liked to see more participation from the class”
Inter-group Interaction	“[Thriving Together could benefit from] some opportunity to switch groups and meet other people” “[Thriving Together could be further improved by] switching tables during the event, so we can chat with more than one group of people for the evening! I still feel like I meet new faces in my class everyday, and the more people to socialize with, the better.” “I didn't like the single-group aspect”
Follow Up	“I really appreciated having a session on culture change and the importance of it. However, we weren't given any resources to use if we identify an unhealthy class culture…” “Could this be a series throughout, I enjoyed the session, but feel that the class could benefit from multiple sessions.”

In the 2023 survey, of the 20 respondents, students agreed that class culture has an impact on their educational experience (4.1 ± 0.5). Moreover, in terms of what shapes their expectations for class culture, 17 (85.0%) felt the second-year medical class had a significant influence (through social activities, extracurriculars, and study habits). Although only 31.3% felt that the discussion around collaboration versus competition within the workshop changed their perspective on class culture, respondents identified their class as collaborative to begin with (relative to competitive; 4.1 ± 0.8). Regarding the workshop itself, students felt that Thriving Together would have a lasting positive impact on class culture (3.7 ± 0.4), and 89.5% expressed wanting similar workshops at other transition points in medical school (e.g., clerkship, residency). 

All respondents recommended the workshop to future cohorts of students, with several elements emerging from narrative responses generally praising the workshop. Students felt that the opportunity to discuss the transition to medical school with classmates was a major benefit of the workshop. Moreover, there was considerable reference to how the workshop helped students feel better supported - both from classmates and from upper-year students.

In terms of how Thriving Together could be further improved, the qualitative responses fell into several main themes. These themes included attendance, inter-group interaction, and follow. Attendance emerged as a primary theme during analysis, with many students endorsing that broader participation could improve the workshop’s impact and allow for more perspectives during small group discussions. With this, the emphasis was on disseminating the workshop in the hopes of reaching more students within the cohort (e.g., shortening the length to make it more accessible; and making it part of the formal curriculum). Furthermore, inter-group interaction was identified as a key theme for improvement. Students expressed interest in mixing groups during the workshop to allow attendees to interact with more students. One respondent stated that they did not like the single-group aspect. The final theme identified through qualitative analysis revolved around follow-up after the event. Participants indicated the need for formal tools and follow-up materials to support future culture change and collaboration outside of the workshop. Illustrative quotes of these themes are included in Table 1.

## Discussion

Tuckman and Jensen’s model of group development describes four main stages of team development: Forming, Storming, Norming, and Performing [24]. Forming is the initial phase of team building, which is closely followed by “storming” where conflicts and competitiveness can arise. Norming occurs when members get to know each other and agree on systems to follow. Finally, “performing” is characterized by effective collaboration in a group setting [24].

The Canadian Interprofessional Health Collaborative (CIHC) framework focuses on developing competencies such as team functioning, collaborative leadership, and communication that will promote interprofessional teamwork in healthcare settings. While Interprofessional Education (IPE) initiatives have been successful at incorporating the CIHC framework into Canadian Medical School curricula, there is a lack of research exploring initiatives that aim to increase intra-cohort collaboration within medical schools. Thriving Together seeks to bridge this gap.

Drawing on Tuckman and Jensen’s model of development, Thriving Together works to decrease the intensity of the “Storming” phase and encourage the progression to the “Performing” phase by introducing a formal “Norming” phase. This is achieved through the workshop, where class dynamics and culture are discussed early in medical training. Through these efforts, a collaborative and supportive environment is fostered at the start of medical training which is essential for combating the demanding nature of medical school [25,26].

This quality improvement study aimed to evaluate the effectiveness of the workshop and identify areas for improvement. Overall, the results demonstrate a positive view of the workshop and an interest in its continuation. However, the results also suggested areas of improvement. Below, the results are interpreted, potential adjustments for future workshops are highlighted, and recommendations are suggested to improve the impact of Thriving Together moving forward.

Attendance

The rate of voluntary attendance has been high across workshops, averaging 48.6%. Over 90% of students who attended would recommend Thriving Together to future students and were interested in a follow-up session, highlighting the workshop's effectiveness despite its voluntary nature. Although students felt that they were able to interact with a variety of classmates, participants indicated that broader class participation would be valuable. Strategies to increase attendance could include integration into the core undergraduate medical curriculum, scheduling adjustments, enhanced promotion, or increased attendance incentives. Identifying and reducing barriers to attendance could increase participation. Furthermore, incorporating Thriving Together into mandatory curriculum could ensure broader participation, and consistent impact year over year.

Workshop design

Qualitative attendee comments highlighted the benefits of meeting new classmates and interacting with upper-year facilitators during the workshop, emphasizing its positive impact on class culture and collaboration. Given that the workshop is intentionally held early on in the first year of medical school, the timing of the workshop appears to be optimized to lend to new connections being made during the workshop.

While randomization of the groups aimed to diversify interactions, participants felt that this could be furthered with inter-group conversations. Implementing activities or opportunities for students to switch groups during the workshop could enhance interactions and encourage broader connections.

Follow-up

Students expressed interest in formal resources on class culture. Developing and distributing materials such as case studies related to organizational change and conflict resolution strategies could offer support for students beyond the workshop. Providing students with these resources may sustain changes to class culture. In addition to formal resources, students were interested in formal follow-up sessions after the initial workshop during key transition points in their career, such as the start of clerkship or prior to residency. Transition to Residency (TTR) courses have been shown to increase confidence and clinical skills, but students still experience a sense of unpreparedness for residency [27]. The incorporation of Thriving Together or similar initiatives could improve collaboration during these transitions.

Limitations

The primary limitation of this study is the small sample sizes of both the workshop attendees and the survey response rate. With an average attendance of less than half the class, the results used to evaluate Thriving Together may not be fully reflective of the entire cohort. These limitations are largely attributed to the voluntary nature of the workshop. Voluntary attendance and survey completion contribute to the potential for response bias, impacting the generalizability of this study. Incomplete data from the satellite campus in Saint John New Brunswick limits the ability to assess the workshop’s impact across multiple locations. As this study was conducted at Dalhousie University, the unique class culture, institution, and composition of students likely impacted the results. It’s important to note that generalizing these results to larger or more diverse cohorts or populations may be limited. To improve generalizability, further studies with a broader scope including other medical schools are required. Furthermore, the study lacks long-term follow-up which is essential for assessing the sustained impact on class culture and collaboration.

Despite these limitations, the study has several strengths including an in-depth analysis of firsthand feedback from Canadian medical students who attended the workshop. The findings highlight the value of the Thriving Together workshop and provide insight into improving collaborative learning environments in medical school. Beyond that, the workshop’s format also offers the potential for student-led initiatives to contribute to a more supportive and collaborative class culture in medical school.

## Conclusions

The Thriving Together workshop has had a positive impact on class culture at Dalhousie Medical School, as evidenced by positive feedback and high rates of recommendation to incoming students. Feedback suggests value in increasing attendance rates, enhancing intra-group interaction during the workshop, and offering post-workshop resources. To improve future workshop attendance, strategies such as scheduling adjustments, increased incentives, and incorporating the workshop into the mandatory curriculum will be considered for future workshops. Additionally, shuffling groups during the session to diversify interactions and formal resources on organizational change and team dynamics will be implemented.

Continuous improvement of the workshop aims to meet the evolving needs of first-year medical students. By addressing the areas for improvement, the Thriving Together workshop will continue to facilitate fostering a collaborative class culture and supportive learning environment for medical students. Future research hopes to explore the long-term impacts of the workshop and the potential to integrate similar workshops at key transition points in medical education such as clerkship and residency.

## Appendices

**Table 2:** Thriving Together 2022 workshop survey questions

1. Would you be interested in a follow-up session in the Spring? (yes/no)
2. Can you describe what you expected or hoped for in this workshop? (narrative response)
3. Did the workshop meet your expectations? (yes/no)
4. What was missing or what would you like to have seen included more? (narrative response)
5. Would you recommend Thriving Together to a friend in next year’s Med 1 class? (yes/no)
6. Were there any negative effects of attending Thriving Together? (yes/no)
7. Can you explain the benefit(s) or the downside(s) of the experiences, in your own words? (narrative response)
8. Are there follow-up experiences or outcomes you would like to see your class take on now and into the future? (narrative response)
9. What support would you need for further class cohesion and effectiveness in the future?
10. Who should provide these supports/initiatives? Options include (check all that apply): i. Informal class leaders ii. Class council iii. Dalhousie Medical Student Society (DMSS) iv. Student affairs in curricular sessions v. DalMedWell non-curricular new programs vi. Other (narrative responses permitted)
11. Would you access self-directed skill-building experiences or resources about: i. Evidence-based findings about what qualities or components are demonstrated by successful groups or teams (yes/no) ii. Understanding the personal emotional and interpersonal qualities that shape your role in a group? (yes/no) iii. Examples of respectful direct interpersonal communication approaches? (yes/no) iv. Case studies of group experiences in health professionals in particular? (yes/no) v. Conflict resolution strategies? (yes/no) vi. Personal and interpersonal effectiveness growth? (yes/no) vii. Other ideas?
12. Please rank in order from 1-5 how you would like to best access information or experiences: i. Directed reading on your own; ii. Facilitated small group sessions; iii. Brief video resources; iv. Near-to-peer led presentations and discussion (for example, with resident mentors); v. Two-day interactive workshop/conference
13. Anything else you’d like to reflect, suggest, or tell us?

**Table 3:** Thriving Together 2023 Workshop Survey Questions

1. In one word, how would you describe your class? (narrative response)
2. How would you define the term “class culture”? (narrative response)
3. How much do you think class culture impacts your educational experience? (Likert Scale of 1, “Not at all”, to 5, “Very much so”)
4. How would you describe the overall culture of your class? (Likert Scale of 1, “Very negative” to 5, “Very positive”)
5. How do you think the workshop impacted your class culture? (Likert Scale of 1, “Very negatively” to 5, “Very positively”)
6. How would you rate the level of competition versus collaboration within your class? Likert Scale of 1, “Highly competitive” to 5, “Highly collaborative”)
7. Did the discussion about collaboration versus competition during the workshop change your perspective on your class culture and your medical school experience going forward? (narrative response)
8. Do you find your expectations for your class culture are influenced by how other years are interacting as a class? (social activities, extracurriculars, study habits) (yes/no; narrative response)
9. Did you have any actionable items or goals that you left Thriving Together with? If so, please share (i.e., seat scramble, different events, study plans) (narrative response)
10. Would you like to have/or have liked to have similar Thriving Together events at other transition points in your medical school experience (i.e., transition to second year, clerkship, residency) (yes/no)
11. What would you change about Thriving Together? Is there anything you feel could be improved or other topics you would like addressed? (narrative response)
12. Would you recommend this event to a friend? Why or why not? (yes/no; narrative response)
13. Do you have any suggestions about how the overall social experience in medical school can be improved for future incoming first-year students (not limited to the workshop itself)? (narrative response)
